# Robo1 and vimentin regulate radiation-induced motility of human glioblastoma cells

**DOI:** 10.1371/journal.pone.0198508

**Published:** 2018-06-04

**Authors:** Pascaline Nguemgo Kouam, Günther A. Rezniczek, Anja Kochanneck, Bettina Priesch-Grzeszkowiak, Thomas Hero, Irenäus A. Adamietz, Helmut Bühler

**Affiliations:** 1 Institute for Molecular Oncology, Radio-Biology and Experimental Radiotherapy, Ruhr-Universität Bochum, Medical Research Center, Marien Hospital Herne, Herne, Germany; 2 Department of Obstetrics and Gynecology, Ruhr-Universität Bochum, Medical Research Center, Marien Hospital Herne, Herne, Germany; 3 Department of Radiotherapy and Radio-Oncology, Ruhr-Universität Bochum, Medical Research Center, Marien Hospital Herne, Herne, Germany; University of South Alabama Mitchell Cancer Institute, UNITED STATES

## Abstract

Glioblastoma is a primary brain tumor with a poor prognosis despite of many treatment regimens. Radiotherapy significantly prolongs patient survival and remains the most common treatment. Slit2 and Robo1 are evolutionarily conserved proteins involved in axon guidance, migration, and branching of neuronal cells. New studies have shown that Slit2 and Robo1 could play important roles in leukocyte chemotaxis and glioblastoma cell migration. Therefore, we investigated whether the Slit2/Robo1 complex has an impact on the motility of glioblastoma cells and whether irradiation with therapeutic doses modulates this effect. Our results indicate that photon irradiation increases the migration of glioblastoma cells *in vitro*. qPCR and immunoblotting experiments in two different glioblastoma cell lines (U-373 MG and U-87 MG) with different malignancy revealed that both Slit2 and Robo1 are significantly lower expressed in the cell populations with the highest motility and that the expression was reduced after irradiation. Overexpression of Robo1 significantly decreased the motility of glioblastoma cells and inhibited the accelerated migration of wild-type cells after irradiation. Immunoblotting analysis of migration-associated proteins (fascin and focal adhesion kinase) and of the epithelial-mesenchymal-transition-related protein vimentin showed that irradiation affected the migration of glioblastoma cells by increasing vimentin expression, which can be reversed by the overexpression of Slit2 and Robo1. Our findings suggest that Robo1 expression might counteract migration and also radiation-induced migration of glioblastoma cells, a process that might be connected to mesenchymal-epithelial transition.

## Introduction

Gliomas represent 30 to 40% of all intracranial tumors. They are categorized as grade IV tumors in the classification of the World Health Organization (WHO) [1]. Approximately half of all gliomas in adults are glioblastomas [2,3]. Radiation therapy plays an important role in the treatment of these tumors, next to surgery and systemic chemotherapy. To date, radiotherapy is the most effective treatment option, prolonging patient survival by several months [4,5]. While there have been many attempts at further optimizing the results of radiation therapy, improvements in patient survival and in local control of tumor growth have not materialized. Among these attempts were dose escalations beyond 60 Gy as well as boost saturation and the use of several radio-sensitizing substances [4].

The main problem of glioblastoma treatment is the high recurrence rate. Renewed tumor growth occurs in the margin of the operated area and/or of the irradiated volume [5]. However, recurrences can also be observed at greater distance from the primary tumor as well as within the treated tissue volume [1]. These observations suggest that treatment failure is caused by diverse mechanisms. Tumor cell migration may play a decisive role in the case of relapses at the margins, which occur at rates of up to 90% [6], but also in remote tumor growth. Migration also complicates cytotoxic therapy because migrating cells are less frequent than non-migrating cells in the dividing phase, in which they are sensitive to cytotoxic medication [2,7,8]. Concerning the influence of ionizing radiation on the motility of glioblastoma cells, the literature provides only scarce and highly contradictory information. Wild-Bode et al. [8] reported an increase in migration and invasiveness after radiotherapy, while Kleynen et al. [9] observed reduced migration after irradiation. Our own observations after low-dose photon irradiation of glioblastoma cells in vitro showed increased motility [10]. As the target volume within the sensitive brain substance has to be limited as much as possible, such an increase in cell motility induced by radiotherapeutic doses could severely hamper an effective local treatment of these tumors.

The Slit/Robo system is an evolutionarily conserved ligand/receptor system usually resulting in chemo-repulsion, which is involved in axon guidance, axonal branching, and the regulation of neuronal cell migration during the development of the central nervous system [11–15]. The binding of the ligand Slit2 to its receptor Robo1 is accompanied by a change in the degree of Robo1-oligomerization, which entails conformation changes in the cytosolic domain. As a result, binding sites for intracellular effectors become vacant. As effector proteins bind to the different intracellular cc-motives of Robo1, the actin cytoskeleton is reorganized and, thus, actin polymerization and cell migration are regulated [14,16,17].

Deletions or epigenetic modifications in the genes for Slit2 and Robo1 have been ascertained in numerous cancer types. In many different carcinomas, such as colorectal, lung, kidney, and mammary carcinoma, the promoter for Slit2 is mostly hypermethylated [18]. The same applies to tumors in the brain, such as neuroblastoma, Wilms’ tumor, glioma cell lines and primary tumors [19–22]. All of these malignant tumors are characterized by a lower level of Slit2 expression than is present in non-neoplastic tissue [19,23]. In addition, the Robo1 receptor is supposed to have a tumor suppressive effect. Mice lacking Robo1 develop invasive lung adenocarcinoma [23–27]. The Slit2/Robo1 (or Robo2) system is involved in the guided migration of breast cancer cells and is suspected to mediate their metastasis into the brain [28].

It is assumed that Slit and Robo play an important role in the migration of metastasizing cells and in their capacity to invade tissues. In 2008, Mertsch et al. reported that the Slit2/Robo1 system is involved in the cell migration of gliomas [22]. According to Yiin et al., Slit2 inhibits cell invasion by gliomas [29]. We therefore decided to investigate to what extent Slit2/Robo1 regulates the motility of glioblastoma cells and if this system could influence radiation-induced migration. We used cultivated human glioblastoma cells in our research. The results showed that an overexpression of Robo1 reduced the natural rate of migration of these cells and was furthermore able to suppress the radiation-induced increase in migration. The epithelial-mesenchymal transition (EMT)-associated structural protein vimentin appears to be involved in these effects.

## Materials and methods

### Cell lines

U-87 MG and U-373 MG cells were obtained from the American Type Culture Collection (ATCC, Manassas, VA, USA) and cultivated at 37°C and 5% CO_2_ in Dulbecco’s modified Eagle’s medium (DMEM, Cat.No. FG0445), supplemented with fetal calf serum (FCS, 10%; Cat.No. S0115), penicillin/streptomycin (100 U/ml and 100 μg/ml, respectively; Cat.No. A2213) (all reagents were from Merck/Biochrom, Berlin, Germany).

To establish stable clones expressing full-length Slit2 (Genbank accession number NM_004787.1; U-87 MG-Slit2, U-373 MG-Slit2) or Robo1 (NM_133631.3; U-87-Robo1, U-373 MG-Robo1), corresponding cDNA constructs were first inserted into the multiple cloning site of pIRESneo (Clontech), a mammalian expression vector featuring the CMV immediate early promoter that drives expression of a transcript containing an internal ribosomal entry site which permits the translation of two reading frames, the cDNA of interest and the neomycin phosphotransferase (allowing for G-418 selection), via SmaI and EcoRV restriction sites (both plasmids were verified by sequencing), transfection of XhoI-linearized plasmids (control: plasmid without insert) into the respective cell line using Lipofectamine LTX (ThermoFisher, Darmstadt, Germany), selection with G-418 (250 U/ml; Cat.No. A2912, Merck/Biochrom), and expansion of individual resistant colonies. Expression of the respective mRNAs and proteins was verified by qPCR analyses and immunoblots, respectively, and clones with adequate expression used for further experiments.

### Colony formation assay

To determine clonogenic survival after irradiation, the glioblastoma cell lines U-373 MG and U-87 MG were treated with photon doses of 0 to 8 Gy. One day before irradiation, 150 cells in 3 ml medium were disseminated per well of standard 6-well tissue culture plates and incubated at 37°C and 5% CO_2_. After irradiation, the cells were incubated under identical conditions until most of the colonies in the control sample (non-irradiated cells) had reached a size of more than 50 cells. Then, the cells were fixed with freezing-cold methanol for 3 minutes. The fixed cells were then stained with crystal violet for 45 minutes. Colonies >50 cells were counted under a light microscope.

### RNA interference assay

U-373 MG and U-87 MG cells were transfected with 80 pmol Robo1 small interfering RNA (siRNA; Cat.No. sc-42252, Santa Cruz Biotechnology, USA) or mock siRNA (control sample; Cat.No. sc-37007) using Lipofectamine LTX following the kit’s instructions. After 72 hours, the knockdown of Robo1 was verified by immunoblot analysis.

### Cell irradiation

Cells were suspended in 2 ml of appropriate medium, seeded in 3-cm culture dishes, and incubated for one day at 37°C and 5% CO_2_. Then, the cells were irradiated with photons using a Synergy S linear accelerator (16 MeV; Elektra, Hamburg, Germany) at the Department of Radiotherapy and Radio-Oncology (Marien Hospital Herne). During irradiation, the cells were kept at room temperature. The doses were 0.5 Gy, 2 Gy, and 8 Gy at a dose rate of 5 Gy/min. The non-irradiated control samples (0 Gy) were treated identically (transport, cultivation).

### Video-microscopic analysis of cell migration

An established videography system [30] was used to investigate cell migration. The system allows observing cell migration over defined periods under normal cell-culture conditions (37°C, 5% CO_2_). Cells (10,000 to 16,000) were seeded in a 3-cm-culture dish and were incubated at 37°C and 5% CO_2_. After 24 hours, the medium was refreshed and the cells were incubated for another 2 to 3 hours. Subsequently, the culture dish was placed into the videography system’s culture chamber and the cells were photographed in 10-minute intervals over 24 hours. Image series were processed and analyzed with ImageJ (http://rsbweb.nih.gov/ij/) or a custom tracking software (sciTaxis; G. Rezniczek; available upon request). Tracking data was further evaluated using Chemotaxis and Migration Tool 2.0 (http://www.ibidi.com).

### Cell adhesion assay

Glioblastoma cell lines were detached with Trypsin/EDTA, washed with medium, and resuspended at 1×10^6^ cells/ml. Cells were incubated with 5 μM calcein AM (Cat.No. 4892-010-K; R&D System, Wiesbaden, Germany) at 37°C and 5% CO_2_ for 30 minutes. The stained cells were washed three time with medium, resuspended at 1×10^5^ cells/ml, and 10,000 cells were seeded per well of a 96-well-plate (at least 10 replicates). On the next day, after a medium change, baseline fluorescence was measured in a plate reader (TECAN Infinite M200, Männedorf, Switzerland; excitation: 494 nm, emission: 517 nm). Then, cells were washed with PBS and incubated with 100 μl of either 0.05% trypsin/0.02% EDTA (w/v) or 0.5 mM EDTA alone for various durations (60–120 seconds) at room temperature, with gentle rocking on an orbital shaker (45 rpm) during the last 20 seconds. Serum-containing medium was added, and detached cells were removed by aspirating the supernatant and gently adding 100 μl of medium twice. The fluorescence of the remaining cells was measured. An adhesion index was calculated as remaining fluorescence divided by baseline fluorescence and expressed as percentage.

### Protein isolation and immunoblot analysis

To isolate proteins from monolayer cell cultures, medium was aspirated and cells washed with phosphate-buffered saline (PBS), and subsequently lysed in 1x Roti-Load sample buffer (Carl Roth, Karlsruhe, Germany) with additional homogenization using an ultrasonic probe (Misonix, Farmingdale, NY, USA). Lysates were incubated at 90°C for 5 minutes and cleared by centrifugation (1 minute, 10.000 g). Protein lysates were separated using SDS-8%-PAGE and blotted onto nitrocellulose membranes (Schleicher & Schüll, Dassel, Germany) in a tank blot unit (Mini-PROTEAN II, BioRad, Hercules, CA, USA). After blocking with a 3% BSA solution, membranes were incubated with primary antibodies reacting with: Robo1 (1:1000; Cat.No. ab7279), fascin (1:3000: Cat.No. ab78487) (abcam, Cambridge, UK), vimentin (1:1000; Cat.No. 9782), focal adhesion kinase (FAK; 1:1000; Cat.No. 3285), Phospho-FAK (Tyr925; 1.1000; Cat.No. 3284) (Cell Signaling Technology, Frankfurt, Germany), and β-actin-POD (1:25000; Cat.No. A3854, Sigma-Aldrich, St. Louis, USA). HRP-conjugated secondary antibodies (Cell Signaling Technology) and the LumiLight plus Western Blotting Substrate (Roche Diagnostics, Mannheim, Germany) were used. Chemiluminescence was recorded using the ChemiDoc MP system and Image Lab program (Bio-Rad, München, Germany).

### qPCR analysis

Total RNA was isolated from cultivated cells using the Total RNA Isolation NucleoSpin RNA II kit (Cat.No. 740955.250, Macherey-Nagel, Düren, Germany) and according to the instructions provided with the kit. The QuantiTect Reverse Transcription kit (Cat.No. 205311, Qiagen, Hilden, Germany) was used to synthesize cDNA from 1 μg RNA. Diluted cDNA (4 μl, corresponding to 6.7 ng of the original RNA) was used in 10-μl-PCR reactions, further consisting of 5 μl 2x QuantiTect SYBR Green buffer (Cat.No. 244052, Qiagen, Hilden, Germany) and 1 μl 10x primer mix. Primers used were ROBO1 (Hs_ROBO1_2_SG; Cat.No. QT01668982), SLIT2 (Hs_SLIT2_1_SG; Cat.No. QT00007784), and GAPDH (Hs_GAPDH_2_SG; Cat.No. QT01192646) (all from Qiagen). Samples were run in triplicates on a 7900HT real-time PCR system (Applied Biosystems, Darmstadt, Germany). Data were analyzed using the SDS software (Applied Biosystems). In each sample, expression levels were normalized using the mRNA expression of the housekeeping gene GAPDH.

### Statistical analysis

GraphPad Prism (GraphPad Software, La Jolla, CA) was used for data analysis (Student’s t-test, Mann Whitney U test).

## Results

### Irradiation leads to increased motility of glioblastoma cells

**Fig 1:**
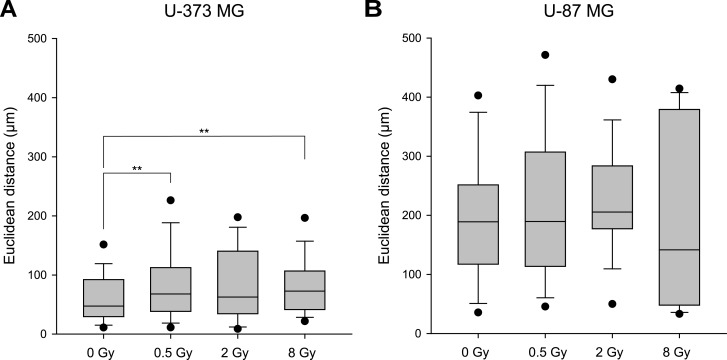
Motility of irradiated glioblastoma cells. U-373 MG (A) und U-87 MG (B) cells were irradiated with doses between 0 Gy and 8 Gy. After irradiation, the cells were observed using time-resolved videography for 24h and the motility parameter Euclidean distance was determined. Each experiment was repeated at least four times. Box plots: the lines in the boxes represent the medians; boundaries, whiskers, and filled circles indicate the 25th/10th/5th (75th/90th/95th) percentiles, respectively. **p<0.01 (Mann-Whitney U test).

We investigated the influence of photon irradiation on the migration of the two glioblastoma cell lines U-373 MG und U-87 MG. Irradiation led to an increase in cell motility as shown by the parameter Euclidean distance. The effect was greatest at low radiation doses of 0.5 Gy and 2 Gy and for U-373 MG (Fig 1).

A substantial increase in motility after photon irradiation was observed in U-373 MG wild-type cells (Fig 1A). The effect was greatest after the lowest dose (0.5 Gy). Compared to the non-irradiated control samples, the mean Euclidean distance was increased from (59.8 ± 4.5) μm to (89.3 ± 8.3) μm (p<0.01; n = 104). Higher radiation doses of 2 Gy and 8 Gy also increased motility to Euclidean distances of (81.83 ± 8.73) μm (n = 60) and (81.94 ± 6.64) μm (p<0.01; n = 62), respectively. In contrast, irradiation of U-87 MG wild-type cells hardly led to significant increases in motility, and after a high dose of 8 Gy even to a slight reduction (Fig 1B). The measured mean Euclidean distances were (197.65 ± 18.56) μm for non-irradiated cells and (218.26 ± 21.84) μm (p = n.s.; n = 70), (222.97 ± 16.31) μm (p = n.s.; n = 70), and (190.42 ± 45.07) μm (p = n.s.; n = 68) for cells irradiated with 0.5 Gy, 2 Gy, and 8 Gy, respectively.

### Increased migration correlates with low expression of SLIT2/ROBO1 in glioblastoma cells

**Fig 2:**
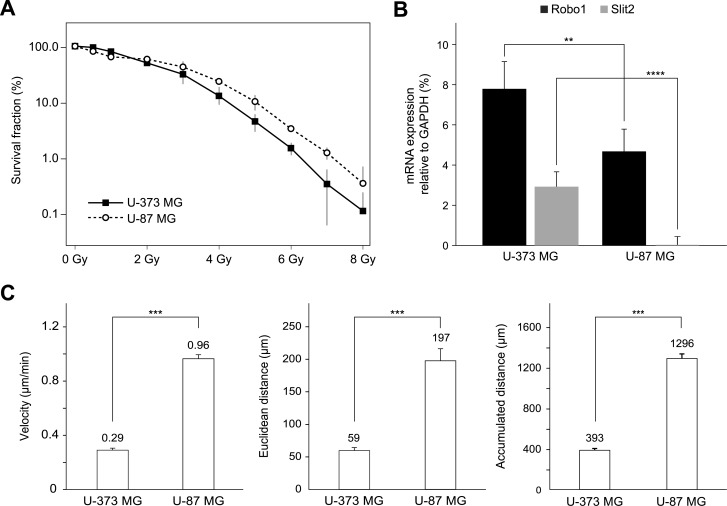
The increased motility of U-87 MG cells compared to U-373 MG cells appears to correlate with a low expression of Robo1 and Slit2. (A) Survival curve of glioblastoma cells after irradiation. The cells were irradiated with up to 8 Gy. (B) Quantification of Robo1-, Slit2-mRNA expression by qPCR. Values represent means ± standard deviation and are relative to the expression of the housekeeping gene GAPDH (100%). ****p<0.0001, **p<0.01 (Student t-test). (C) Motility of non-irradiated U-373 MG and U-87 MG cells. Cell motility was measured by the parameters velocity, Euclidean distance, and accumulated total distance. Data shown are means ± standard error. ***p<0.001 (Mann-Whitney U test).

We were able to show that there is a correlation between radiation resistance (Fig 2A), increased motility, and a lower grade of Slit2 and Robo1 expression. Using time-resolved videography, it was possible to quantify the migration of the two glioblastoma cell lines U-373 MG and U-87 MG and to correlate it with the level of expression of Slit2/Robo1 (Fig 2B). Cells of the U-373 MG line, which express both Robo1 and Slit2 (Fig 2B), moved at a much slower rate (ca. 0.29 ± 0.014) μm/min than those of the U-87 MG cell line (0.96 ± 0.029) μm/min. The accumulated distance traveled by U-87 MG cells was almost three times as long as that covered by U-373 MG cells (Fig 2C). U-87 MG cells exhibited a low Robo1 expression, which was about. 40% lower than that of U-373 MG cells, but Slit2 was hardly expressed in them.

Based on these observations, we were wondering whether the observed considerable increase in motility of U-373 MG cells upon irradiation might be related to the expression levels of Slit2 and Robo1. Next, we therefore quantified their mRNA expression after irradiation with different doses.

### Irradiation modulates Robo1 mRNA expression in U-373 MG cells

**Fig 3:**
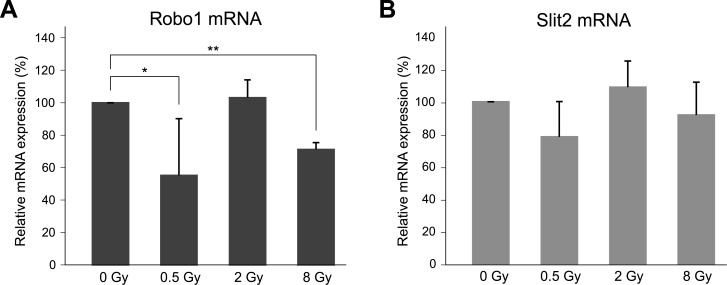
Effect of irradiation on the expression of Slit2 and Robo1 in U-373 MG cells. Robo1 (A) and Slit2 (B) mRNA in U-373 MG cells quantified by qPCR 24h after photon irradiation. Relative expression levels were calculated using the ΔΔCt method (reference gene: GAPDH) and are shown relative to those in the non-irradiated controls (0 Gy, 100%). Data represent means ± standard deviation. **p<0.01, *p<0.05 (Student t-test).

We irradiated U-373 MG cells with different doses (0.5 Gy, 2 Gy, and 8 Gy) and after 24 hours compared their levels of Slit2 and Robo1 mRNA expression with those in non-irradiated control cells (Fig 3). Robo1 mRNA expression was significantly decreased after low-dose (0.5 Gy) or high-dose (8 Gy) irradiation to approx. 50% and 70%, respectively, but no significant effect was observed after irradiating with 2 Gy (Fig 3A). Changes in Slit2 mRNA levels, while not significant, resembled the Robo1-results (Fig 3B).

### Overexpression of Robo1 and Slit2 reduces the motility of U-373 MG cells

**Fig 4:**
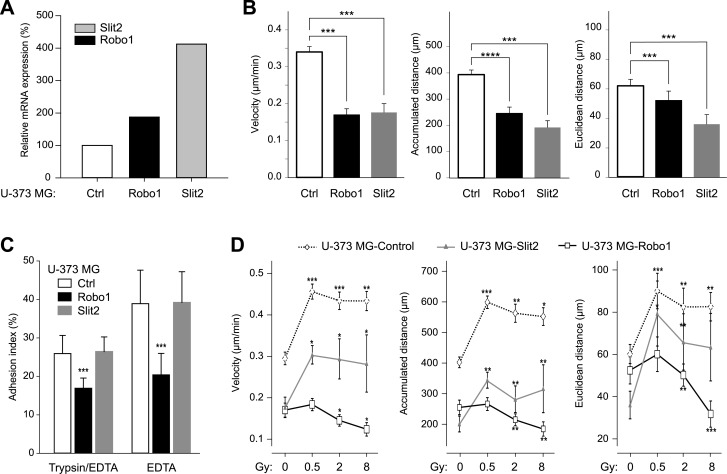
Effect of Slit2 and Robo1 overexpression on U-373 MG cell motility and adhesion. (A) Quantification of Robo1 and Slit2 mRNA expression in Robo1 and Slit2 overexpressing clones of the cell line U-373 MG. Relative expression levels were calculated using the ΔΔCt method (reference gene: GAPDH) and are shown relative to control (100%). Ctrl, U-373 MG-Control (control cells transfected with the empty vector). (C) Quantification of the adhesion of U-373 MG cells overexpressing Slit2 or Robo1 compared to that of control cells. Cells were treated either with trypsin/EDTA or EDTA alone. The adhesion index represents the percentage of adherent cells after the detached cells were removed. (B and D) Effect of irradiation and/or overexpression of Slit2 and Robo1 on the motility of U-373 MG cells. The motility of non-irradiated U-373 MG-Control, U-373 MG-Robo1, and U-373 MG-Slit2 cells (B, 0 Gy in D) or after irradiation with doses of 0.5 Gy, 2 Gy, and 8 Gy was compared. The cells were observed for 24h using time-lapse video microscopy and the motility parameters velocity, accumulated distance, and Euclidean distance were determined. Data represent means ± standard errors from at least four separate experiments. ****p<0.0001, ***p<0.001, **p<0.01, *p<0.05 (B and D, Mann-Whitney U test; C, ANOVA/Tukey).

Transfection of Robo1 or Slit2 expression cassettes into U-373 MG resulted in clones with ~1.8-fold (U-373 MG-Robo1) and ~4.8-fold (U-373 MG-Slit2) overexpression of the respective mRNAs (Fig 4A). In both clones, we observed significantly decreased cell motility. Control cells typically traveled a distance that was twice as long as the one traveled by the overexpressing clones: (393.4 ± 17.3) μm versus (246.1 ± 23.7) μm (p<0.001; n = 95) and (191.9 ± 26.0) μm (p<0.001; n = 95) in case of U-373 MG-Robo1 and U-373 MG-Slit2, respectively (Fig 4B). Velocity was correspondingly reduced in both transfected clones to approx. 60% of the velocity measured in the control cells: (0.34 ± 0.01) μm/min versus (0.169 ± 0.01) μm/min (p<0.001; n = 95; U-373 MG-Robo1) and (0.176 ± 0.02) μm/min (p<0.001; n = 95; U-373 MG-Slit2), respectively. Regarding Euclidean distance, the overexpression of Slit2 led to a similar reduction: (36.1 ± 6.5) μm versus (62.05 ± 4.46) μm in control cells. However, the reduction was not as pronounced in the U-373 MG-Robo1 clone with (52.2 ± 6.3) μm. To determine whether the reduced motility of Slit2 or Robo1 overexpressing cells was due to a putative increase in their adhesion capacity, we performed an adhesion assay where adherent cells were exposed to either a combination of the serine protease trypsin and EDTA or EDTA alone. As expected, the enzymatic treatment was more effective. While there was no difference in adhesion between control and Slit2-overexpressing cells, U-373 MG-Robo1 (as well as U-87 MG-Robo1, data not shown) cells exhibited significantly greater sensitivity (Fig 4C).

### Overexpression of Robo1, but not of Slit2, inhibits the irradiation-induced increase in cell motility

We also investigated if the irradiation-induced increase in motility of U-373 MG cells could be modulated through the overexpression of Slit2 or Robo1. For this purpose, the overexpressing clones as well as the control cells were photon-irradiated with doses of 0.5 Gy, 2 Gy, and 8 Gy. Similar to the control cells, U-373 MG-Slit2 cells exhibited an increased motility after irradiation. This effect was greatest after a dose of 0.5 Gy, and only slightly weaker after doses of 2 Gy or 8 Gy (Fig 4D). Compared to non-irradiated U-373 MG-Slit2, a dose of 0.5 Gy led to an increase of 70% in velocity [from (0.18 ± 0.02) μm/min to (0.30 ± 0.02) μm/min (p<0.05; n = 14)], of 74% in the total distance covered [from (191.9 ± 26.0) μm to (335.1 ± 26.0) μm (p<0.001; n = 14)], and of 49% in Euclidean distance [from (36.1 ± 6.5) μm to (78.8 ± 15.2) μm (p<0.05; n = 14)]. Thus, overexpression of Slit2, while reducing the overall motility of such cells compared to control cells, did not affect the relative changes in motility that are induced by irradiation with different doses. In contrast to this, U-373 MG-Robo1 cells showed only a slight increase in cell motility after irradiation with a dose of 0.5 Gy, whereas higher doses of 2 Gy and 8 Gy even decreased motility in these cells (Fig 4D). After irradiation with a dose of 0.5 Gy, U-373 MG-Robo1 cells showed, in comparison with their non-irradiated counterparts, an increase in velocity by 7% [from (0.17 ± 0.02) μm/min to (0.18 ± 0.01) μm/min], in distance covered by 4% [from (246.1 ± 23.7) μm to (257.0 ± 21.1) μm], and in Euclidean distance by 9% [from (52.2 ± 6.3) μm to (59.8 ± 8.1) μm]. In summary, overexpression of Robo1 in U-373 MG cells did not only decrease baseline levels of cell motility but also inhibited the otherwise observed irradiation-induced increase in motility.

### siRNA-knockdown of Robo1 increases cell motility

**Fig 5:**
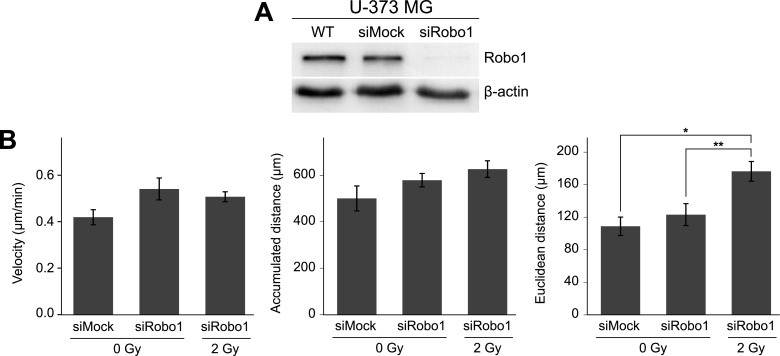
Motility of U-373 MG cells after Robo1-downregulation by siRNA. (A) Western blot assay demonstrating the downregulation of Robo1. WT, untreated control cells; siMock, siRobo1, cells transfected with control or Robo1-specific siRNAs. (B) The motility parameters velocity, accumulated distance, and Euclidean distance were determined in non-irradiated (0 Gy), Robo1-knockdown, and control U-373 MG cells and in Robo1-knockdown cells irradiated with a dose of 2 Gy. Data represent means ± standard errors. **p<0.01, *p<0.05 (Mann-Whitney U test).

In order to further test if Robo1 is indeed involved in regulating the motility of (irradiated) glioblastoma cells, we have diminished the expression of Robo1 through transfection of a specific siRNA (Fig 5A). The effect of the knockdown of Robo1 on the motility of non-irradiated U-373 MG cells or of cells irradiated with 2 Gy was then analyzed using time-lapse videography (Fig 5B). Judged by all three motility parameters (velocity, cumulative and Euclidean distance), Robo1-knockdown cells exhibited a slight increase in motility, ranging between 20%, 15% and 13% (p = n.s.). Irradiation caused a further increase in motility, specifically for the Euclidean distance covered by these cells [(176.4 ± 12.11) μm] versus non-irradiated cells [(123.2 ± 13.48) μm; p<0.047] or cells transfected with mock siRNA [(108.9 ± 11.36) μm; p<0.01].

### Forced overexpression of Robo1 reduces the motility of the aggressive and radiation-resistant glioblastoma cell line U-87 MG

**Fig 6:**
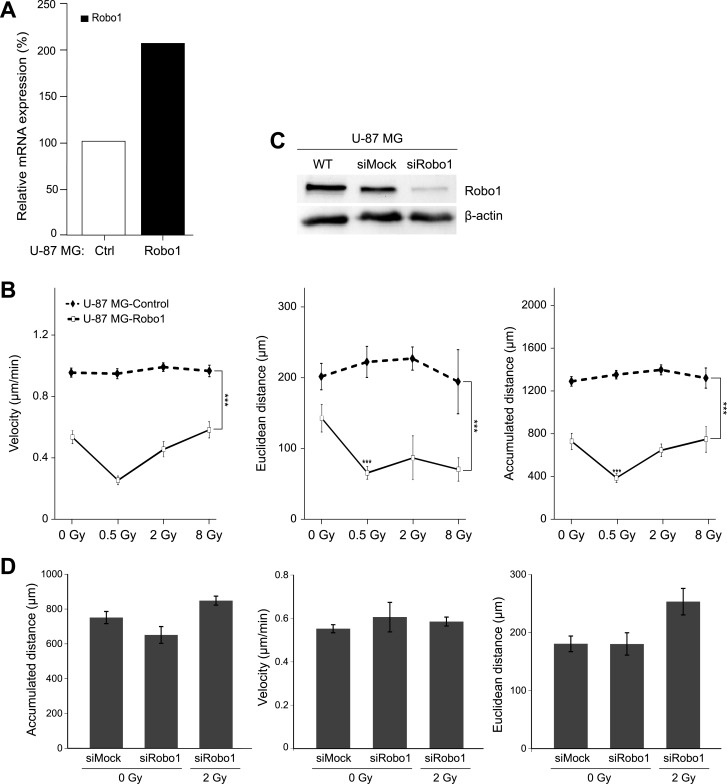
Effect of Robo1 overexpression on U-87 MG cell motility. (A) Quantification of Robo1 mRNA expression in U-87 MG-Control cells (Ctrl) and U-87 MG-Robo1 cells (Robo1). Relative expression levels were calculated using the ΔΔCt method (reference gene: GAPDH) and are shown relative to control (Ctrl, 100%). (B) Motility of U-87 MG-Control and U-87 MG-Robo1 cells 24h after photon irradiation with doses ranging from 0 Gy to 8 Gy. Motility of the Robo1-overexpressing cells (solid line) is lower than that of wild-type cells (dashed line). (C) siRNA-knockdown of Robo1 in U-87 MG cells as demonstrated by Western blot analysis. (D) The motility parameters velocity, accumulated distance, and Euclidean distance were determined in non-irradiated (0 Gy) Robo1-knockdown and control U-87 MG cells and in Robo1-knockdown cells irradiated with a dose of 2 Gy. Data (B and D) represent means ± standard errors. ***p<0.001 (Mann-Whitney U test).

Overexpression of Robo1 (Fig 6A) significantly reduced the motility of radiation-resistant U-87 MG cells, which are known to be characterized by increased migration and invasion and which are also resistant to radiation.

Compared to the control cells, Robo1-overexpressing cells (U-87 MG-Robo1) travelled at a reduced speed of (0.54 ± 0.042) μm/min versus (0.97 ± 0.03) μm/min (p<0.001; n = 54) and only covered approx. half the distance [from (1296.3 ± 44.8) μm down to (735.4 ± 76.4) μm (p<0.001; n = 54)]. The Euclidean distance was lowered to (141.66 ± 19.41) μm from (197.65 ± 18.56) μm (p<0.05; n = 54) (Fig 6B).

Irradiation of U-87 MG-Robo1 cells resulted in an interesting effect (Fig 6B). Using a radiation dose of 0.5 Gy, all of the motility parameters (velocity, accumulated distance, and Euclidean distance) were diminished to about half compared to non-irradiated cells [velocity: from (0.54 ± 0.23) μm/min to (0.26 ± 0.12) μm/min (n = 77); accumulated distance: from (735.4 ± 76.4) μm to (392.5 ± 41.5) μm (n = 75); Euclidean distance: from (141.7 ± 19.4) μm to (64.8 ± 8.9) μm (n = 65); all: p<0.001]. In contrast, radiation doses of 2 Gy and 8 Gy had no significant effect on velocity and accumulated distance of these cells compared to non-irradiated U-87 MG-Robo1. However, the Euclidean distance remained on the same low level as observed in U-87 MG-Robo1 cells irradiated with 0.5 Gy [(85.8 ± 31.0) μm and (69.7 ± 16.4) μm] (Fig 6B).

The effect of the knockdown of Robo1 (Fig 6C) on the motility of non-irradiated U-87 MG cells or cells irradiated with 2 Gy was also analyzed using time-lapse videography. The motility parameters (velocity, cumulative and Euclidean distance) were slightly increased for Robo1-knockdown cells (Fig 6D).

### The reduction in radiation-induced migration through Robo1 and Slit2 appears to be mediated by a lowered expression of vimentin, which may imply an MET-process

**Fig 7:**
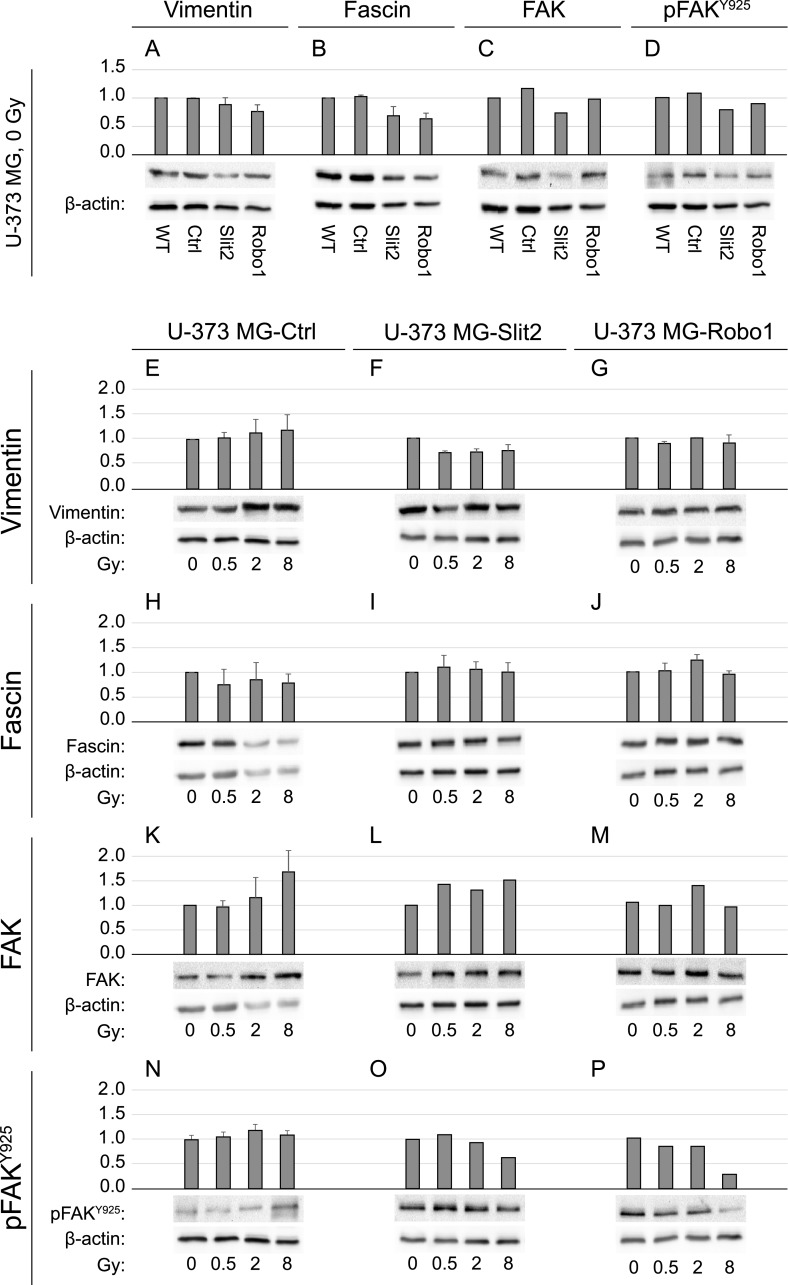
Semiquantitive immunoblot analyses. The expression of vimentin (A, E-G), fascin (B, H-J), focal adhesion kinase (FAK) (C, K-M), and phosphorylated focal adhesion kinase (pFAK^Y925^) (D, N-P) was quantified in U-373 MG wild-type cells and clones overexpressing Slit2 or Robo1 and the effect of photon irradiation on their expression was assessed. Proteins were isolated 24h after irradiation. WT, U-373 MG wild-type cells; Ctrl, U-373 MG control cells (transfected with empty vector); Robo1, U-373 MG-Robo1 (stable clone overexpressing Robo1); Slit2, U-373 MG-Slit2 (stable clone overexpressing Slit2).

In order to find out by which mechanism the proteins Robo1 and Slit2 regulate radiation-induced motility of glioblastoma cells, we investigated the expression of the migration-associated proteins fascin and FAK/pFAK^Y925^ and the EMT marker protein vimentin. Immunoblotting analyses show that irradiation increases the expression of vimentin in wild-type cells (Fig 7E). This expression was slightly reduced in non-irradiated cells overexpressing either Slit2 or Robo1 (Fig 7A), and remained lower than in wild-type cells after irradiation (Fig 7F and 7G). Irradiation of wild-type cells (Fig 7H) and a Slit2 or Robo1 overexpression reduced the expression of fascin (Fig 7B). In addition, we observed an increase or no change in the expression of this protein in clones after irradiation (Fig 7I and 7J). We found an increased expression of FAK in the wild-type cells as well as in the clones after irradiation (Fig 7K–7M). An overexpression of Slit2 and Robo1 had no significant influence on the expression of FAK (Fig 7C). Irradiation of wild-type cells increased the activation of FAK through phosphorylation at tyrosine 925 (Fig 7N). The forced expression of Slit2 or Robo1 reduced the activation of FAK (Fig 7D) and maintained its level under ionizing radiation in case of Slit2-overexpressing cells (Fig 7O) but caused a reduction in the Robo1-overexpressing cells (Fig 7P). A dose of 8 Gy reduced phosphorylation in both overexpressing clones.).

## Discussion

Notwithstanding a possible resection, radiotherapy is the most frequent treatment for glioblastoma. The prognosis for this type of tumor has remained poor because of a high recurrence rate in the immediate vicinity of the resection or radiation target volume. It is possible that the relapses are influenced by the tumor cell’s ability to migrate and to invade tissue as tumor cells increasingly separate themselves from the tumor region and infiltrate the surrounding healthy tissue, where they form metastases. This process lowers the effectiveness of radiotherapy. Therefore, this study investigated the effect of irradiation on the migration of cultivated glioblastoma cells. Dependent on the dose, we found different effects of irradiation on the motility of the analyzed cell lines. In U-87 MG und U-373 MG wild-type cells, irradiation increased migration, and this effect was greatest when a relatively low radiation dose of 0.5 Gy or 2 Gy was used (Fig 1). Wild-Bode et al reported already 15 years ago an increase in migration of glioma cells after irradiation with a sublethal dose. They observed a continually rising cell migration after irradiation with 1.3 Gy as well as 6 Gy [8]. While our present study shows such an increase in the motility of glioblastoma cells after irradiation with 0.5 Gy and 2 Gy, we also found that a higher dose of 8 Gy had a weak or even slightly inhibiting effect on motility. Göetze et al. came to a similar result. They, too, observed an increase in migration after irradiation with 1 Gy and 3 Gy and a reduction after a dose of 10 Gy [31]. In a former project, we observed an increase of migration in U-251 MG cells after irradiation with a dose of 2 Gy, which, however, could be inhibited through a treatment with hyperbaric oxygen [10]. In summary, a dose from 0.5 Gy to 6 Gy increases the migration of glioblastoma cells, whereas doses from 8 Gy to 10 Gy have the opposite effect. It is a well-known fact that ionizing radiation causes numerous types of damage to the DNA, thus leading to genomic instability, potential tumorigenesis, and cell death [32]. Damage to the DNA is assumed to occur in proportion with the applied radiation dose [33,34]. This may explain the decreased migration of cells irradiated with larger doses. However, it remains unclear why low radiation doses cause an increase in migration. A possible answer is that cells surviving these doses change phenotypically with consequences for migration.

To identify the mechanism that causes this effect of radiation, we focused our investigation on the Slit/Robo system. The Slit gene family (Slit1, Slit2, and Slit3) consists of large, extracellular, secreted and membrane-associated glycoproteins that regulate axon guidance, axon branching, and neuronal migration during the development of the central nervous system, and were identified as ligands of receptors of the Robo family [11]. A tumor-repressive activity has been attributed to Slit2, and the Slit2 promoter region is hypermethylated in primary lung cancer, breast cancer, and colorectal carcinomas [18,35]. A frequently occurring Slit2 deactivation through hypermethylation of CpG islands in the promoter region has been observed in glioma cell lines and in glioma tumors. In general, such a hypermethylation correlates with the deactivation of the gene. It was shown that a treatment with a demethylating agent restored transcription and the corresponding protein expression [18]. In case of the Robo1 gene too, hypermethylation was found in breast and kidney cancer cells [25]. Using qPCR, the mRNA expressions of Slit2 and Robo1 were quantified in two glioblastoma cell lines (U-373 MG and U-87 MG) with different malignancy characteristics regarding migration and radiation resistance (Fig 2), and the results were compared. The expression of Slit2 and Robo1 was the lower, the more radiation resistant and the more motile a cell line was. Previous data show that Slit2 is clearly expressed in normal cerebral neurons, whereas it is expressed only to a low degree in pilocytic astrocytomas, fibrillary astrocytomas, and in glioblastomas compared to the normal cortex [22]. Moreover, the expression of Slit2 decreases over time or is even shut down with the progression of the malignant glioma, as Dallol et al. have shown for the cell lines U-87 MG and U-373 MG in comparison with normal brain tissue [18,36]. Thus, our data confirm the results for Slit2 presented in the literature. A reduced expression of Robo1 has been found in prostate cancer [26]. The expression of this protein varies significantly on the level of proteins depending on the glioblastoma cell line and the grade of the glioblastoma. Mertsch et al. investigated the expression of Robo1 on the protein level in 37 gliomas. 75% of the grade-IV glioblastomas, 23.1% of grade-II, and 58.3% of grade-I glioblastomas tested positive for the expression of Robo1. Their study, however, does not present any data on the total amount of the expression. In addition, the authors observed a reduction of Robo1 on the mRNA level in glioblastoma of all grades compared to the cortex. In addition, the immunohistochemical staining showed a higher expression of Robo1 in the neurons of the cortex than in glioblastoma tissue [22]. In a controversial report, Liu et al. described a slightly increased Robo1 expression in the glioblastoma of 40 patients compared to normal brain tissue [36]. Thus, the expression of Robo1 in glioblastoma remains uncertain and further studies are required.

Our analyses show that certain levels of photon irradiation reduce the expression of Slit2 as well as Robo1. Two reasons indicate that Slit2 and Robo1 are potentially involved in the observed increase in migration after irradiation: firstly, the interaction between Slit2 and its receptor Robo1 has an immediate effect on the reorganization of the actin cytoskeleton and thus regulates axon guidance and, therefore, cellular migration; and secondly, irradiation appears to influence the expression of both proteins. Therefore, we assessed the motility of Slit2 or Robo1 overexpressing glioblastoma cells. A substantial decrease down to 50% in the motility of U-373 MG clones was observed compared to wild-type cells (Fig 4). As this cell line per se expresses Slit2 and Robo1, it is possible that an overexpression of Slit2 in the cells leads to an increased Slit2/Robo1 interaction, possibly inhibiting the induction of cell motility due to its neuronal repulsive action. This finding agrees with the results of Yiin et al. who were able to show in a mouse model that the infiltration of SNB19 glioma cells in the surrounding brain tissue was inhibited if the invasive SNB19 cells were implanted with an exogenous expression of Slit2 [29].

Irradiation led to dose-dependent cell-line-specific changes in motility as well as Slit2 and Robo1 expression. To determine if the regulating effect of Slit2 and Robo1 can be modified by radiotherapy, we analyzed the migration of Robo1- and Slit2-transfected cells after irradiation. Depending on the radiation dose, the motility of the analyzed Robo1-overexpressing glioblastoma cell lines remained unchanged or decreased compared to a non-irradiated control sample. In U-87 MG-Robo1 cells, motility decreased significantly after irradiation with doses of 0.5 Gy and 8 Gy. A significant reduction in motility was also observed in U-373 MG-Robo1 cells, particularly after irradiation with 8 Gy. As already mentioned, a Robo1 overexpression lowered the motility of both cell lines by up to 50%. This effect was unchanged 24 hours after irradiation. The motility increase in wild-type cells after irradiation with 0.5 Gy and 8 Gy was inhibited through overexpression of Robo1 in the cell lines U-87 MG and U-373 MG (Figs 4 and 6). In addition, cells transfected with siRNA targeting Robo1 showed an increase in cell motility without irradiation as well as after irradiation with a dose of 2 Gy (Figs 5 and 6D). Recombinant Slit2 was demonstrated to induce a repulsive signal on glioblastoma cells in a Boyden chamber assay. This repulsive effect was canceled out by siRNA-mediated knockdown of Robo1 [22]. In contrast, other studies reported the reduction of migration after a knockdown of Robo1 [36–38]. These findings contradict our results. It has to be noted, however, that the system we used for measuring motility differs significantly from the ones used in other studies. While most researchers used Boyden chambers or scratch tests for measuring migration, we observed the free migration of cells under a microscope. Neither Boyden chambers nor the scratch test (wound healing) measure migration as such, but rather mixed forms of migration including chemotaxis and cell proliferation. Our method makes it possible to observe the free movement of individual cells and, therefore, to assess their potential for locomotion on a support [10,39].

Radiation-induced changes in motility of glioblastoma cells on the one hand and Robo1- und Slit2-mediated motility changes on the other are potentially regulated by different mechanisms. While the overexpression of Robo1 was shown to reduce motility, and this effect was sustained even under irradiation, the overexpression of Slit2 led in contrast to a cell-line-specific reduction in motility, which was partly reversed by irradiation. In the next step, we investigated the mechanism by which Slit/Robo differentially modulate the motility of glioblastoma cells after irradiation. To this end, we examined whether the diminished migration of the clones was due to the changes in their adhesion potential or rather changes to the activation state of key factors in the cellular migration machinery, such as FAK [40]. While we did not observe a difference in the adhesion of Slit2-overexpressing cells compared to wild-type cells, we found that overexpression of Robo1 led to reduced adhesion which could not directly explain the diminished motility of these cells. On the other hand, the overexpression of Robo1 reduced activated FAK, specifically upon irradiation. Previously, our group showed that an increased phosphorylation of FAK correlates with increased migration of mammary carcinoma cells and that both can be prevented with the treatment with zoledronic acid [39]. Our analyses show that irradiation increases the expression of migration-associated proteins such as FAK and fascin in wild-type cells as well as in clones. A strong expression of fascin was found in glioma cells in comparison to normal astrocytes, and a correlation was observed between the overexpression of fascin and a higher grading of glioblastoma tumors, which implies a worse prognosis [41]. Thus, a knockdown of fascin reduces the migration and invasion in glioblastoma cells [42]. It has also been shown that irradiation increases the expression of FAK and therefore the migration of tumor cells including glioblastoma cells [43]. Our results confirm those present in the literature.

In addition, we were able to show that irradiation increases the expression of vimentin in wild-type cells. An overexpression of Robo1 and Slit2 reduced the expression of vimentin and this expression remained lower than in the wild-type cells even after irradiation (Fig 7). Vimentin is an EMT-associated protein and may potentially play a role in cell migration [44]. In recent years, an EMT induction through ionizing radiation was reported which is supposed to increase cell migration and invasion [43,45]. Thus, ionizing radiation led to an increase in the expression of vimentin [46] and to an increased migration of esophageal squamous cancer cells. The present study describes this effect for glioblastoma cells. It appears therefore plausible to assume that both, irradiation-induced migration and the reduction in migration by Slit2 and Robo1 are regulated by an EMT mechanism that here manifests itself in the regulation of vimentin expression.
